# SARS-CoV-2 mRNA Vaccine Breakthrough Infections in Fully Vaccinated Healthcare Personnel: A Systematic Review

**DOI:** 10.3390/tropicalmed7010009

**Published:** 2022-01-13

**Authors:** Caterina Ledda, Claudio Costantino, Giuseppe Motta, Rosario Cunsolo, Patrizia Stracquadanio, Giuseppe Liberti, Helena C. Maltezou, Venerando Rapisarda

**Affiliations:** 1 Occupational Medicine Unit, Department of Clinical and Experimental Medicine, University of Catania, 95123 Catania, Italy; vrapisarda@unict.it; 2 Department of Health Promotion, Mother and Child Care, Internal Medicine and Medical Specialties “G. D’Alessandro”, University of Palermo, 90133 Palermo, Italy; claudio.costantino01@unipa.it; 3 Occupational Medicine Unit, “Garibaldi” Hospital of Catania, 95123 Catania, Italy; giusmotta@gmail.com; 4 Hospital Health Management, “G. Rodolico-San Marco” Polyclinic University Hospital, 95123 Catania, Italy; r.cunsolo@ao-ve.it; 5 Occupational Medicine Unit, “G. Rodolico-San Marco” University Hospital, 95123 Catania, Italy; patrizia.stracquadanio@ao-ve.it; 6 Commissioner Office in Acta for the COVID-19 Emergency, Provincial Health Authority of Catania, 95123 Catania, Italy; commissario.covid@aspct.it; 7 Directorate for Research, Studies and Documentation, National Public Health Organization, 15123 Athens, Greece; maltezou.helena@gmail.com

**Keywords:** mRNA-1273, BNT162b2, TAK-919, COVID-19, SARS-CoV-2, post-vaccination, healthcare personnel, pandemic, vaccination, asymptomatic infection

## Abstract

The number of people vaccinated against COVID-19 increases worldwide every day; however, it is important to study the risk of breakthrough infections in vaccinated individuals at high risk of exposure such as healthcare personnel (HCP). A systematic literature review (SLR) applying the PRISMA declaration and the PECOS format using the following entry terms was used: “Health Personnel OR Healthcare Worker OR Healthcare Provider OR Healthcare Personnel AND breakthrough OR infection after vaccine*”. The research was carried out utilizing the following databases: SCOPUS, PubMed, Embase, and Web of Sciences. An overall very low incidence of post-vaccination breakthrough infections was found, ranging from 0.011 to 0.001 (per 100 individuals at risk). Our findings further support the published high effectiveness rates of mRNA vaccines in preventing SARS-CoV-2 infections among fully vaccinated HCP. Additional studies are needed to define the duration of the vaccine-induced protection among HCP.

## 1. Introduction

Severe acute respiratory syndrome coronavirus 2 (SARS-CoV-2) infections have not been under control in most countries, and the pandemic of coronavirus disease 2019 (COVID-19) continues to be a problem for public health worldwide. As of 10 November 2021, more than 250 million cases and 5 million associated deaths were confirmed [1]. New variants of SARS-CoV-2 strains have emerged, which makes the situation more complex, and new waves recur even in some countries/areas where SARS-CoV-2 infections seemed to be under control [2,3,4].

Various interventions, including mask-wearing, quarantining, and social distancing, have played a significant role in monitoring and regulating the COVID-19 pandemic [4,5,6,7]; nevertheless, vaccination is considered a highly cost-effective intervention to mitigate the pandemic [8,9]. The documented flare-ups and breakthrough cases have been ascribed to three potentials: (1) the circulating variants of SARS-CoV-2 and their effect on vaccine-elicited immunity; (2) the speed of natural decline of antibodies among vaccinated people; and (3) the requirement for booster doses [10,11].

Throughout the past year, COVID-19 vaccines were advanced at an extraordinary speed. As of 10 November 2021, 24 vaccines had been approved by at least one state in the world, 7 vaccines had been authorized for emergency use by the World Health Organization (WHO) [12], and 1 had been fully authorized by the Food and Drug Administration (FDA) [13].

COVID-19 vaccines are created using various technologies: mRNA, protein subunit, inactivated, non-replicating viral vector, and DNA. At first, in December 2020, the United States of America (USA), the United Kingdom, Canada, and the European Union (EU) approved the emergency use of Pfizer-BioNTech’s mRNA vaccine (BNT162b2) [12], and in January 2021, the USA and the EU approved the Moderna mRNA-1273 vaccine [12] through the U.S. FDA and European Medicine Agency, respectively. Today, Pfizer-BioNTech’s vaccine (BNT162b2) is approved in 103 countries, while Moderna mRNA-1273 in 76 countries, the formulation of the latter has recently been marketed by Takeda (TAK-919) only in Japan [12].

In randomized, placebo-controlled, phase 3 efficacy trials after vaccine rollout, both mRNA vaccines were efficient in inhibiting symptomatic and severe COVID-19 illness [14,15,16,17]. Furthermore, a recent study showed that full vaccination of HCP with mRNA vaccine was associated with 66.42% vaccine effectiveness against absenteeism [18]. In clinical trials, the vaccines had 52–95% efficacy for symptomatic disease 14 days later than the first dose and 95% efficacy 7 days later than the second dose [15,16]. Initial observational investigations on mRNA vaccines in healthcare personnel (HCP) indicate 80% effectiveness >14 days following the first dose and 90% >14 days after the second dose [17,19,20]. While the number of vaccinated people increases worldwide, SARS-CoV-2 variants are of interest for their augmented transmissibility, increased disease severity, and immune escaping resulting in the risk of reinfections or breakthrough infections in vaccinated persons [21,22].

Nevertheless, there may be differences in vaccine effectiveness and breakthrough infection rates depending on the timing of testing. Breakthrough infections are mitigated by vaccines and are normally minor in their clinical features [23]. In the first period of vaccination campaigns, institutions had the objective to protect those at the greatest risk of infection because of their high risk of exposure [24]. Therefore, many countries have designated HCP as a main concern group for COVID-19 vaccination [25,26,27]. Therefore, today, HCP are the oldest, largest, and most at risk of vaccine breakthrough infection group. Moreover, little is known about how many infected vaccinated subjects can spread the infection [28,29].

This systematic literature review (SLR) provides evidence on mRNA reported vaccine breakthrough infections among HCP.

## 2. Materials and Methods

### 2.1. Design

An SLR of the records of diverse databases was carried out, subsequent to a pre-set procedure firstly established to minimalize the risk of bias in both choice and publication and safeguarding optimum organization and content. The methodology was to follow the standards specified in the PRISMA declaration [30] and applying the Evidence-Based Health Practice methodology [31], as well as using the Joanna Briggs Inventory (JBI) Checklist for Prevalence Studies tool to evaluate the risk of bias [32]. The instrument was used with the objective of improving consistency in SLR of prevalence records and has been suggested as the most suitable tool for this kind of investigation [33]. The risk of bias was assessed using the nine criteria established by Munn [32]. The level of bias is evaluated by assessing the total sum of criteria with a “yes” reply and transforming this score into a percentage (n/9). Studies obtaining <50% are judged as having high risk of bias, 50–69% medium risk of bias, and ≥70% low risk of bias.

### 2.2. Literature Search

We searched until 10 November 2021 in SCOPUS, PubMed, Embase, and Web of Sciences. The entry terms used were: “Health Personnel OR Healthcare Worker OR Healthcare Provider OR Healthcare Personnel AND breakthrough OR infection after vaccine*”. The examination of the appropriate papers for inclusion in this SLR was also carried out, and the research articles were recovered and reviewed.

### 2.3. Inclusion and Exclusion Criteria

The following inclusion criterion was used: surveys that assessed SARS-CoV-2 mRNA vaccine breakthrough infections in HCP. The exclusion criteria adopted were: (1) animal studies, (2) abstract and case reports, (3) articles that were not available in English. For replicate studies, the article with more detailed data was integrated.

Moreover, investigations assessed as high risk of bias were excluded from the primary analysis.

### 2.4. Quality Assessment and Data Extraction

Two reviewers (C.L. and V.R.) studied the manuscripts separately. The title, abstract, and full text of each potentially relevant manuscript were reviewed. Any concerns regarding suitability of the manuscript were defined through consensus. The following data were investigated from all included papers: study design, country, mRNA vaccine, period observed, and breakthrough incidence of fully vaccinated HCP. If incidence was not expressed in the study, it was calculated by the authors. The breakthrough was proven by the detection of SARS-CoV-2 found by Reverse-Transcriptase Polymerase Chain Reaction (RT-PCR) test in swab samples.

## 3. Results

### 3.1. Characteristics of Eligible Studies

**Figure 1:**
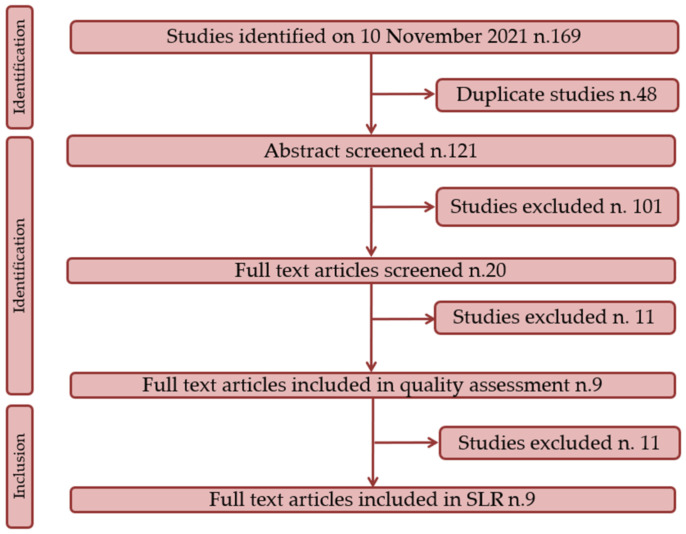
Graph illustrating included and excluded studies in the SLR.

Following a search of the relevant databases, 121 documents were identified. Of these, 101 were excluded after review of the title and abstract, and 7 studies were excluded after review of the manuscript. After all, nine studies satisfied the inclusion criteria and were included in the SLR [34,35,36,37,38,39,40,41,42]. A flow-chart depicting the studies selected in the present SLR is shown in Figure 1.

### 3.2. Results of Eligible Studies

All the studies reviewed investigated COVID-19 infection in HCPs after the emergency vaccination campaign. In detail, seven studies included HCP vaccinated with both the BNT162b2 vaccine and the mRNA-1273 vaccine were conducted in the USA, and only one was conducted in Belgium [17,35,36,37,39,40,41]. The other studies were in Israel and Greece using the BNT162b2 vaccine [25,29]. The studies covered a period from 9 December 2020 to 14 August 2021. The breakthrough incidence varied from a minimum of 0.001 to a maximum of 0.011. Table 1 summarizes the features of the studies analyzed.

Bergwerk et al. [34], to evaluate the effectiveness of the BNT162b2 vaccine, carried out, through a prospective cohort study of 11,453 HCP, a case control investigation among 1497 fully vaccinated HCP. In this study, researchers reported the positivity to SARS-CoV-2 by RT-PCR in 39 fully vaccinated HCP. The mean age of the 39 sick workers was 42 years, and most of them were women (64%). The median period from the second vaccine dose to SARS-CoV-2 finding was 39 days (range, 11 to 102). Only one sick person (3%) was immunosuppressed. Moreover, in 37 cases of breakthrough infection, the suspected source of infection was an unvaccinated person. Of all the HCP with breakthrough infection, 26 (67%) had mild symptoms at various stages, and none needed hospitalization. The residual 13 workers (33% of all cases) were asymptomatic throughout the period of infection. The most frequent symptom that was described was higher respiratory congestion (36% of all cases), subsequent myalgia (28%), and loss of smell or taste (28%); fever or rigors were registered in 21% of participants.

A prospective cohort study was carried out by Bouton and colleagues [35]. They observed a total of 10,590 HCP, but only 5913 had obtained 2 doses of the vaccine (BNT162b2 or mRNA-1273) at that time. In only 17 HCP did post-vaccination cases of SARS-CoV-2 occur. Another prospective cohort study performed in the USA [36] observed 4136 HCP with no earlier laboratory-documented SARS-CoV-2 disease for 35 weeks. Of these, 2976 were fully vaccinated (BNT162b2 or mRNA-1273), and 34 HCP reported the infection of SARS-CoV-2, 80.6% of which were symptomatic.

In Belgium, Geysels et al. [37], in a prospective cohort study of 3491 fully vaccinated (BNT162b2 or mRNA-1273) HCP, 9 workers (0.3%) were positive for SARS-CoV-2 RT-PCR test. Of the nine HCP who were fully vaccinated, five were vaccinated with the BNT162b2 vaccine, and four were vaccinated with the mRNA-1273 vaccine.

Ioannou et al. [38] compared viral load, clinical report at diagnosis, and type of exposure between vaccinated (1800) (with BNT162b2) and non-vaccinated (450) HCP. Among all 55 PCR-positive HCP, 21 were fully vaccinated (diagnosed >2 weeks later than the second dose). Interestingly, the viral load did not differ significantly between vaccinated and non-vaccinated HCP; nevertheless, the kind of symptoms differed significantly. Specifically, rhinorrhea and nasal congestion were significantly more common in vaccinated HCP, while cough and fever were more frequent in non-vaccinated HCP.

A large survey was performed in the USA by Jacobson and colleagues [39] from December 2020 to April 2021 involving 22 271 HCP fully vaccinated with mRNA-based SARS-CoV-2 vaccine. Among these, 26 cases of SARS-CoV-2 occurred in fully vaccinated HCP. The mutation types of breakthrough infections were: 0 (0%) E484K, 10 (55.6%) L452R, N501Y 1 (5.6%), and no mutation 7 (38.9%). Again in the USA, North et al. [40] in a prospective cohort study of 2243 fully vaccinated HCP, observed three infections, among which only one was symptomatic.

Teran [41] reported 22 cases of postvaccination SARS-CoV-2 diseases among skilled nursing facility residents and HCP. Among the 22 individuals with breakthrough infections, 14 (64%) were asymptomatic. Three symptomatic persons had mild, imprecise symptoms; two had mild, certain symptoms; and three had diagnosed pneumonia, one of these, with basic conditions of hypertension, diabetes mellitus, and chronic kidney disease, died.

Lastly, Thompson [42] performed a prospective cohort study including 2686 HCP who received two doses of SARS-CoV-2 mRNA vaccine. SARS-CoV-2 infection was discovered in five fully vaccinated HCP. The authors provided the vaccine effectiveness that was 92% (80–97; 95% CI) among fully vaccinated persons. In particular, the vaccine effectiveness was 94% (82–98; 95% CI) and 84% (31–96: 95% CI) for the BNT162b2 vaccine and the mRNA-1273 vaccine, respectively.

## 4. Discussion

The present SLR analyzed SARS-CoV-2 breakthrough infections that occurred in HCP fully vaccinated with mRNA SARS-CoV-2 vaccine. Studies included in this systematic review reported a very low incidence (0.001 to 0.011 per 100 individuals at risk) of post-vaccination reinfection among HCP in the first six months following the primary vaccination cycle [25,26,27,28,29,30,31,32,33]; the death of a single fully vaccinated individual due to COVID-19 breakthrough infection was reported [41]. Vaccine effectiveness on HCP analyzed by this systematic literature review remains high and constant between different countries.

Prior to the kickoff of anti-SARS-CoV-2 vaccination, HCP were the group with the maximum risk of exposure to COVID-19 infection [43,44]. Gholami et al., in a systematic review and meta-analysis, proved that the proportion of HCP who confirmed positive for COVID-19 between 28 surveys was 51.7%, with a 15% rate of hospitalization and a 1.5% death rate [43]. Although breakthrough infections mean that the virus broke through a protective barrier provided by the vaccine, it prevents COVID-19 in more than 90% of beneficiaries [14,15]. Recent investigations carried out among HCP fully vaccinated by mRNA vaccine and continuously examined by routine nasal testing have demonstrated significant decreases, but not a total absence, of SARS-CoV-2-positive tests [17,19,20,34,35,36,37,38,39,40,41,42]; in detail, almost all of the HCP were asymptomatic and a near absence of hospitalizations was reported, and some case reports just reported the infection among vaccinated HCP [44,45,46,47,48,49].

Ioannou and colleagues [38] compared the viral loads among vaccinated and unvaccinated HCP and did not find statistically significant differences as regards age, gender, site of acquisition, occurrence of symptoms at diagnosis, and viral loads. His findings, however, are opposite to those found in other studies with stronger enrollment. Thompson et al. [42] showed that among HCP with SARS-CoV-2 infection, the mean viral RNA load was 40% lower (95% CI, 16 to 57) in incompletely or fully vaccinated persons rather than in unvaccinated people. A survey among nursing home residents infected by SARS-CoV-2 and vaccinated with only a single dose of BNT162b2 evidenced that nasopharyngeal viral load was lower in vaccinated people [28].

A comparable outcome was described by Levine-Tiefenbrun et al. [50] in the analysis of a real-world dataset of COVID-19 patients after vaccination by the BNT162b2 mRNA vaccine; they found that the viral load was significantly decreased for infections following 12–37 days after the first dose of vaccine.

Therefore, since the viral load is correlated to transmission [51,52], single-dose mRNA SARS-CoV-2 vaccination might prevent outbreaks. Reduced viral loads indicate a potentially decreased infectiousness, further than reducing vaccine impact on virus spread.

The evaluation of viral loads is critical in the control of breakthrough infections and the management of SARS-CoV-2 variant outbreaks. Jacobson et al. found a high prevalence of breakthrough infection in HCP due to the L452R variant [39]. A study carried out in Israel observed that B117 is associated with higher viral load and observed higher viral load in B117 variant infections when compared to other variants [53]; moreover, higher viral loads in B1351 infections in unvaccinated individuals compared to fully vaccinated persons were reported [54].

An augmented percentage of variants in vaccine breakthrough infections that appears in two distinct windows of period have been reported: The first augmented quantity of B.1.351 was discovered in patients fully vaccinated with BNT162b2, 7–14 days after the second dose, matched to unvaccinated controls. Moreover, an augmented percentage of B.1.1.7 was detected in partially vaccinated persons 14 days after the first dose up to 6 days after the second dose [53].

The behavior of the variants was also studied in in vitro neutralization tests that demonstrated a significant decrease in neutralization against B1351 and a small reduction against B117 in fully vaccinated persons [55,56,57,58].

Many seroprevalence studies on SARS-CoV-2 have been conducted. At first, they were carried out to estimate the prevalence among HCP [59,60,61,62]. After vaccination, several studies were carried out in order to provide indications on the presence of antibodies after vaccination and the dosages were repeated over time [63,64,65,66,67]. mRNA vaccines stimulate anti-spike IgG in addition to T cell reactions that can be discovered in peripheral blood [68]; however, how long immunity is stimulated by SARS-CoV-2 mRNA vaccine is even under investigation at this phase. Tretyn and colleagues [69] analyzed the components of the immune response in vaccinated persons. After mRNA vaccination, the values of the humoral response were detected in the whole of people enrolled, which verified the effectiveness of the mRNA vaccine in triggering B lymphocytes to release antibodies and T lymphocytes to secrete interferon-γ [69].

Doria-Rose et al. [70] based on ad hoc phase 3 trials of the Moderna mRNA-1273 estimated the half-life of vaccine-binding antibodies and defined the lifetime of the vaccine immune response at 6 months after second dose. Bayart and colleagues [71], in a multicenter prospective study, focused on longer-term kinetics information of the humoral response following the two-dose regimen of BNT162b2 mRNA vaccine and found a significant antibody decrease 6 months post-vaccination. The decrease was highly significant for overall antibodies, IgG, and neutralizing antibodies in both seronegative and seropositive participants. Thus, the clinical implications of serological assays that were not yet clear from a clinical position and the founding of thresholds connected with defense are still needed, but the association between low neutralizing antibody titers and breakthrough infection may not be excluded and might rationalize the request of appropriate vaccination policies, particularly in HCP, frail patients, and their caregivers [34,72,73,74].

The main limit of the present study could be related to the limited period (less than six months from the completion of primary vaccination cycle) of observation of the studies included in the SLR.

As is well-known, starting from Israel, all European countries, the UK, and the US that began the vaccination campaign among HCP in December 2020–January 2021 have offered the booster doses to HCP since September 2021 (especially those older than 60 years or with at least one comorbidity).

Furthermore, another aspect to underline is the difficulties in the implementation of equitable distribution of anti-SARS-CoV-2 vaccines.

Vaccination has increased slowly worldwide. However, while few high-income countries’ governments understand how to vaccinate their whole populations during the pandemic, most low- and middle-income countries have been trusting the COVID-19 Vaccines Global Access (COVAX) facility to acquire vaccines [75]. COVAX aims to require these countries with adequate doses to vaccinate 20% of their people.

Public vaccine improvement efforts should move in the direction of decreasing all characteristics of public health risk instead than favoring its business financial characteristics [76,77].

Future analyses are needed to evaluate the length of the primary vaccination cycle protection among HCP, also standardized for age and at-risk groups.

## 5. Conclusions

In conclusion, we conducted a systemic review of published evidence on post-vaccination breakthrough SARS-CoV-2 infections among HCP fully vaccinated with mRNA vaccines. An overall very low incidence of post-vaccination breakthrough infections was found, ranging from 0.011 to 0.001 (per 100 individuals at risk). Our findings further support the published high effectiveness rates of mRNA vaccines in stopping SARS-CoV-2 infections including fully vaccinated HCP. Further studies are required to define the duration of the vaccine-induced protection among HCP.

## Figures and Tables

**Table 1:** Features of suitable studies.

Reference	JBI Score	Study Design	Country	mRNA Vaccine	Period Observed	Breakthrough Incidence *
Bergwerk et al. [34]	100%	Case-control study	Israel	BNT162b2	20 January 2021–28 April 2021	0.003
Bouton et al. [35]	88%	Prospective cohort study	United States of America	BNT162b2 or mRNA-1273	9 December 2020–23 February 2021	0.003
Fowlkes et al. [36]	88%	Nested cohort study	United States of America	BNT162b2 or mRNA-1273	14 December 2020–14 August 2021	0.011
Geysels et al. [37]	100%	Prospective cohort study	Belgium	BNT162b2 or mRNA-1273	1 March 2021–30 April 2021	0.003
Ioannou et al. [38]	66%	Prospective cohort study	Greece	BNT162b2	4 January 2021–14 April 2021	0.009
Jacobson et al. [39]	100%	Prospective cohort study	United States of America	BNT162b2 or mRNA-1273	18 December 2020–2 April 2021	0.001
North et al. [40]	100%	Prospective cohort study	United States of America	BNT162b2 or mRNA-1273	30 December 2020–2 April 2021	0.001
Teran et al. [41]	100%	Prospective cohort study	United States of America	BNT162b2 or mRNA-1273	28 December 2020–31 March 2021	0.002
Thompson et al. [42]	88%	Prospective cohort study	United States of America	BNT162b2 or mRNA-1273	14 December 2020–10 April 2021	0.001

* = only in fully vaccinated HCP.
